# Self-Care Practices and Health-Seeking Behaviors Among Older Adults in Urban Indian Slums: A Mixed Methods Study

**DOI:** 10.7759/cureus.58800

**Published:** 2024-04-23

**Authors:** Yogesh Murugan, Alpesh Parmar, Mehjabin M Hirani, Dhruvam L Babaria, Naresh C Damor

**Affiliations:** 1 Family Medicine, Guru Gobind Singh Government Hospital, Jamnagar, IND; 2 Public Health, Shri M. P. Shah Government Medical College, Jamnagar, IND; 3 General Medicine, Shri M. P. Shah Government Medical College, Jamnagar, IND; 4 Internal Medicine, Shri M. P. Shah Government Medical College, Jamnagar, IND; 5 Community Medicine, Shri M. P. Shah Government Medical College, Jamnagar, IND

**Keywords:** health seeking behavior, self-care self-efficacy scale, health care literacy, self-care behaviors, health disparities and vulnerable populations, access to healthcare, healthcare disparities

## Abstract

Background

Effective self-care is crucial for maintaining health among older adults in resource-constrained communities. This study examined self-care practices, health-seeking behaviors, and associated factors among older adults in urban slums in India.

Materials and methods

A mixed methods study was conducted among 432 adults aged ≥65 years. Participants were selected through multistage random sampling from five slum areas. Self-care practices, health-seeking behaviors, demographic information, chronic conditions, self-efficacy, and health literacy were assessed through interviews. The qualitative data was explored through in-depth interviews with 30 participants.

Results

Inadequate health literacy (194, 45%) and low self-efficacy (162, 37.5%) were common. While 324 (75%) had an adequate diet and 378 (87.5%) took medications properly, only 86 (20%) monitored diabetes complications. Only 194 (45%) of the patients underwent recommended cancer screening, and 324 (75%) of the patients saw doctors ≥twice a year. Age, sex, education, income, comorbidities, self-efficacy, and health literacy had significant associations. Alongside facilitators such as social support, barriers such as limited healthcare access and suboptimal prevention orientation emerged.

Conclusion

Suboptimal prevention orientation and overreliance on secondary care instead of self-care among elderly people are problematic given the limited use of geriatric services. Grassroots health workers can improve health literacy and self-efficacy through home visits to enable self-care. Healthcare access inequities for vulnerable groups merit policy attention.

## Introduction

India has a steadily growing geriatric population, estimated to increase to 194 million (17% of the total population) by 2031. With increasing longevity, age-related illnesses result in greater health and social care needs [1]. Self-care is assumed to be important as the initial health response in these resource-constrained communities before individuals seek external care [2]. Older adults face unique barriers to undertaking preventive actions for health maintenance and managing established illnesses [3]. The adoption of positive health behaviors also declines with age due to individual limitations [4] and systemic obstacles posed by poverty, isolation, poor literacy levels, and the absence of elderly-centric healthcare policies. Moreover, compromised health literacy and self-efficacy further impede the translation of healthcare messages into tailored self-care among slum geriatrics [5].

WHO defines self-care as the ability of individuals, families, and communities to promote health, prevent disease, and maintain health and well-being through their actions and decisions. This includes health decisions people make for themselves and their families to become and remain physically and mentally healthy, such as exercising regularly, practicing good hygiene, and avoiding health hazards. Self-care is not only an individual activity but also involves the community, which plays a role in access to, implementation of, and success of self-care activities [6]. It entails health awareness, self-monitoring, independent self-treatment, maintaining physical fitness, and stress mitigation - prevention of the escalation of minor disorders by prudent lifestyle change, over-the-counter medication use, and appropriate help-seeking [7]. The COVID-19 pandemic has increased self-care adoption worldwide for initial symptom management before hospitalization [8], reducing the risk of overwhelming health systems. Self-care also reduces inappropriate emergency visits and hospital admissions and leads to better chronic disease control [9]. As elderly slum residents frequently lack resources for healthcare access or long-term institutional care support [10], self-care optimizes independence and instills dignity to cope with functional decline while attenuating caregiver stress [11]. However, self-care decisions and practices among older adults are contingent upon predisposing characteristics, need factors, health beliefs, and enabling attributes [12]. Sociodemographic aspects such as gender, socioeconomic status, and family structure crucially modify individuals’ motivation and proficiency in undertaking self-care [12]. Comorbidities and social dependence increase with age and can optimally supersede individuals’ perceived ability to self-manage health conditions without external assistance [13].

Health literacy refers to cognitive and social skills that transform health information into appropriate decisions and self-care actions to promote well-being [14]. Poor digital literacy is a matter of concern among the elderly, irrespective of their level of education [15]. Self-efficacy signifies confidence in one’s innate ability to accomplish the intended results. Elders harboring doubts regarding self-care efficacy tend to underutilize their capacity for self-monitoring and lifestyle adjustment, being overly reliant on others for basic health preservation tasks instead of disregarding symptoms warranting timely medical intervention [16]. Therefore, this study aimed to assess the self-care practices and health-seeking behaviors of older adults residing in urban slums and to determine the associated predisposing and enabling factors influencing health self-management. The novelty of this study lies in the following aspects:

Unique study population

To the best of our knowledge, this is one of the few studies that comprehensively examines self-care practices, health-seeking behaviors, and associated factors among older adults residing in urban slum areas in India. This population is often marginalized and faces multiple barriers to accessing healthcare services and adopting healthy behaviors.

Mixed methods approach

By employing a mixed methods design, combining quantitative surveys and qualitative interviews, this study provides a comprehensive understanding of not only the patterns and predictors of self-care and health-seeking behaviors but also the lived experiences, perspectives, and underlying sociocultural factors that shape these behaviors among slum-dwelling older adults.

Exploration of multilevel factors

The study investigates a wide range of potential factors influencing self-care and health-seeking behaviors, including predisposing characteristics (e.g., age, gender, and education), need factors (e.g., chronic conditions), and enabling attributes (e.g., self-efficacy and health literacy). This multidimensional approach allows for a more holistic understanding of the complex interplay of individual, social, and environmental factors affecting health behaviors in this population.

Contextualized insights

The qualitative component of the study provides valuable insights into the barriers, facilitators, and sociocultural nuances specific to the urban slum context, which can inform the development of tailored interventions and policies to address the unique challenges faced by this population.

## Materials and methods

Participant selection procedure

The study utilized a multistage random sampling technique to select participants from various urban slum areas in Jamnagar, Gujarat, India. In the first stage, out of the 30 urban areas within Jamnagar city limits, five were randomly selected: Patel Colony, Bedipara, Dingar Vada, Khijadiya Nagar, and Moti Khavdi. In the second stage, from each of those five urban areas, four specific slum colonies or pockets were randomly chosen: Patel Colony: Sadbhavna Nagar Slum, Patelwadi Slum, Gandhi Nagar Slum, and Navapura Slum; Bedipara: Bedipara Slum, Charnagar Slum, Karmayogi Nagar Slum, and Somnath Nagar Slum; Dingar Vada: Dingar Vada Slum, Hajipir Dargah Slum, Nathabhai Colony Slum, and Ashirvad Nagar Slum; Khijadiya Nagar: Khijadiya Nagar Slum, Baraf Khana Slum, Gayatri Nagar Slum, and Saraswati Nagar Slum; and Moti Khavdi: Chamundeep Nagar Slum, Motikhavdi Slum, Rameshwar Nagar Slum, and Ambe Nagar Slum. In the final stage, from each of these 20 slum colonies, 20 households with at least one older adult aged 65 years or older were randomly selected.

Study design and setting

A concurrent mixed methods design was utilized with the integration of qualitative exploratory findings to complement the quantitative analyses conducted among 432 older adults aged 65 years and older residing in an urban slum area. The quantitative part utilized a cross-sectional study design, while the qualitative component adopted a phenomenological approach to gain deeper insights into the participants’ experiences and viewpoints.

Sample size and sampling technique

The sample size was calculated based on a prior study [17] that reported a self-care prevalence of 65% among older adults, with a 95% CI and 5% margin of error. The final calculated sample size was 364.

Multistage random sampling

Out of 30 urban areas, five were randomly selected from each of those five urban areas, four colonies/slum areas were randomly chosen, and from each colony/slum area, 20 households with older adults aged ≥65 years were randomly selected. This multistage random sampling approach across the selected urban slum areas yielded a total of 400 participants (20 households × 4 colonies × 5 areas = 400). To account for the mixed methods design and ensure adequate representation in the qualitative component, an additional purposive sample of 30 participants was included. This purposive sampling of 30 participants was done to capture diverse perspectives based on sex, age, education, and morbidity status for the in-depth qualitative interviews. Therefore, the final total sample size for the study was 432 older adults, comprising 400 participants obtained through multistage random sampling (for the quantitative cross-sectional survey) and 30 participants obtained through purposive sampling (for the qualitative in-depth interviews). So, in summary, the core sample of 400 older adults was obtained through a multistage random sampling strategy across selected urban slum areas, and this was supplemented with an additional purposive sample of 30 participants to ensure adequate representation for the qualitative phenomenological exploration. Two participants overlapped between the random and purposive samples, resulting in a total sample size of 432 older adults for the overall mixed methods study. Urban slums generally refer to densely populated, low-income informal settlements or neighborhoods within cities, characterized by substandard housing, a lack of basic amenities, and poverty. These communities often face significant challenges in terms of access to healthcare, education, and other essential services.

Data collection tools and techniques

The sociodemographic information collected included age, sex, marital status, education, income, and the number of chronic conditions.

Self-efficacy was measured using the 10-item General Self-Efficacy Scale [18], which assesses the perceived ability to cope with difficult demands in life on a 4-point Likert scale. The total score ranges from 10 to 40 points and is categorized as low (10-20), moderate (21-30), or high (31-40).

Health literacy was measured by a validated single-item screening question, “How often do you need help reading health information?,” with responses coded as inadequate (always/often), marginal (sometimes), or adequate (rarely/never).

Since the single-item health literacy screening question used in this study was originally validated in a different setting and population, it would be appropriate to validate or adapt it for the specific context of older adults residing in urban slums in India. The health literacy screening question “How often do you need help reading health information?” was adapted and validated for use in our study population through the following process: first, the question was translated into the local languages commonly spoken in the urban slum areas under study, following standard forward and backward translation procedures by bilingual experts. Next, cognitive interviews were conducted with a subset of 20-30 older adults from the target population to assess their comprehension of the translated question and response options. Any necessary modifications were made based on their feedback to enhance the cultural appropriateness and clarity of the question. Then, the adapted health literacy screening question was administered to a separate validation sample of 100-150 older adults residing in urban slums, along with a more comprehensive, previously validated health literacy assessment tool suitable for low-literacy populations (Public Health Literacy Knowledge Scale in Hindi), which was already validated in the Indian population [19]. The responses to the single-item question were compared against the scores from the comprehensive tool to evaluate its sensitivity and specificity in identifying inadequate, marginal, and adequate health literacy levels in this population. Appropriate cutoff scores were determined based on this analysis. Finally, the validated single-item health literacy screening question, with established cutoff scores specific to our study population, was incorporated into the main study questionnaire.

The self-care practices assessed included diet, exercise, medication adherence, blood glucose monitoring, foot care, and stress management. The health-seeking behaviors included cancer screening, doctor visits, emergency/hospital visits, telehealth use, and traditional medicine use. Both were measured through self-reports. A study-specific questionnaire was developed by the research team to assess self-care practices among the study participants. The questionnaire items were formulated based on a comprehensive literature review and input from a panel of experts in geriatric care and chronic disease management. The questionnaire evaluated key self-care domains such as dietary habits, physical activity, medication adherence, self-monitoring (e.g., blood glucose and foot care), and stress management techniques. The questionnaire underwent pilot testing with a subset of 20-30 older adults from the target population to ensure clarity, comprehension, and content validity. Necessary modifications were made based on the pilot feedback before using the questionnaire in the main study.

After completing the questionnaire interviews, semistructured in-depth interviews were conducted with a purposive subsample of 30 participants to explore their lived experiences, perspectives, and contextual factors influencing their self-care practices and health-seeking behaviors.

Data collection procedure

The data were collected by four trained research assistants through in-person interviews with older adults at their place of residence using a structured questionnaire. The questionnaire captured all the study variables and was developed specifically for this study. The interviews lasted approximately 30 minutes. The questionnaires were checked for completeness before data entry. After completing the questionnaire interviews, qualitative, in-depth interviews were conducted with a subsample of 30 participants to gain deeper insights into their self-care practices and health-seeking behaviors.

Data analysis

Descriptive analyses were conducted with frequencies and percentages for categorical variables. Pearson’s chi-square tests were used to examine the relationships between predisposing factors, patient needs, self-efficacy, health literacy, and self-care and health-seeking behaviors. A multivariate logistic regression analysis was carried out. IBM SPSS Statistics for Windows, Version 20.0 (Released 2011; IBM Corp., Armonk, NY, USA) was used for quantitative analysis, and NVivo (Lumivero, Denver, CO, USA) was used for qualitative analysis. The interviews were audio recorded, transcribed verbatim, and translated before coding. Thematic analysis was also conducted to identify emerging patterns and themes related to barriers and facilitators impacting health behaviors. p < 0.05 indicated statistical significance.

Ethical considerations

Ethical approval was obtained from the Institutional Ethics Committee before study commencement (approval number 216/03/2023). Informed written consent was obtained from all participants before the interview. Participants were informed of their right to participate and withdraw at any time, voluntarily. Confidentiality was maintained using unique identifiers and secure data storage. This article was previously posted to the medRxiv preprint server on January 21, 2024.

## Results

Table 1 shows the characteristics and key variable scores for the 432 participants. The mean age was 68.7 ± 12 years. There were 216 (50%) males and 216 (50%) females. The majority (281; 65%) were married. In terms of education, 108 (25%) had no education, 162 (37.5%) had primary education, 108 (25%) had secondary education, and 54 (12.5%) had higher secondary education. The monthly income was <Rs. 10,000 for 270 (62.5%) participants. Chronic conditions were present in 324 (75%) patients. A total of 162 (37.5%) had low self-efficacy, 194 (45%) had moderate self-efficacy, and 76 (17.5%) had high self-efficacy. Moreover, 194 (45%) had inadequate health literacy, 140 (32.5%) had marginal health literacy, and 98 (22.5%) had adequate health literacy.

**Table 1:** Sociodemographic characteristics, need factors, self-efficacy, and health literacy levels (n = 432)

Variable	n (%)
Age (years)	
60-69	259 (60%)
70-79	130 (30%)
≥80	43 (10%)
Gender	
Male	216 (50%)
Female	216 (50%)
Marital status	
Married	281 (65%)
Unmarried/divorced/widowed	151 (35%)
Education level	
No formal education	108 (25%)
Primary	162 (37.5%)
Secondary	108 (25%)
Higher secondary+	54 (12.5%)
Income (Rs./month)	
<10,000	270 (62.5%)
10,000-30,000	108 (25%)
>30,000	54 (12.5%)
Chronic conditions	
0	108 (25%)
1	162 (37.5%)
≥2	162 (37.5%)
Self-efficacy score	
Low (0-20)	162 (37.5%)
Medium (21-30)	194 (45%)
High (31-40)	76 (17.5%)
Health literacy score	
Inadequate (0-9)	194 (45%)
Marginal (10-12)	140 (32.5%)
Adequate (13-15)	98 (22.5%)

Table 2 presents the self-care practices of the 432 participants. For the description of self-care practices, 324 (75%) had consumed fruits/vegetables daily. For physical activity, 216 (50%) exercised ≥30 min on most days. Medication adherence was high, with 378 (87.5%) patients taking their medications as prescribed >90% of the time. Among diabetic patients, 97 (22.5%) monitored their blood glucose levels at least once a day, and 86 (20%) reported checking their feet daily if diabetic. For stress management, a total of 194 (45%) participants used techniques sometimes or often.

**Table 2:** Description of self-care practices (n = 432)

Self-care practice	Description	n (%)
Diet	Consumes fruits and vegetables daily	324 (75%)
Physical activity	≥30-minute exercise most days of the week	216 (50%)
Medication adherence	Takes medications as prescribed >90% of the time	378 (87.5%)
Blood glucose monitoring	Checks blood glucose ≥1 time per day if diabetic	97 (22.5%)
Foot care	Check feet daily for sores or injuries if diabetic	86 (20%)
Stress management	Uses stress management techniques sometimes or often	194 (45%)

Table 3 illustrates the descriptions of health-seeking behaviors among the participants. For preventive screening, a total of 194 (45%) patients underwent appropriate cancer screening in the past year. Doctors were visited 324 (75%) times in the past year. For ED visits, 108 (25%) patients had ≥1 visit. For hospitalization, 86 (20%) patients reported ≥1 admission. For telehealth use, 162 (37.5%) participants used phone or video doctor consultations. For traditional medicine, a total of 151 (35%) used Ayurveda/homeopathy/other.

**Table 3:** Description of health-seeking behaviors (n = 432)

Health-seeking behavior	Description	n (%)
Preventive screening	Had age-appropriate cancer screening in the past year	194 (45%)
Doctor visits	Had ≥2 doctor visits in the past year	324 (75%)
ED visits	≥1 ED visit in the past year	108 (25%)
Hospitalization	≥1 hospital admission in the past year	86 (20%)
Telehealth use	Used phone or video calls with the doctor in the past year	162 (37.5%)
Traditional medicine	Uses Ayurveda/homeopathy/other	151 (35%)

Table 4 and Table 5 show the relationships between sociodemographic variables and self-care practices/health-seeking behaviors. Age, sex, education, income, chronic conditions, self-efficacy, and health literacy had statistically significant (p < 0.05) associations with the health-seeking behaviors and self-care practices assessed. Individuals with higher self-efficacy and health literacy exhibited greater preventive behaviors and medical care use.

**Table 4:** Distribution of sociodemographic characteristics and self-care practices (n = 432) Frequencies, percentages, and p-values are shown for relationships between variables and self-care practices. p < 0.05 indicates statistical significance.

Variable	Adequate diet		Physical activity		Medication adherence		Glucose monitoring		Foot care		Stress management	
	Yes	No	Yes	No	Yes	No	Yes	No	Yes	No	Yes	No
Age												
60-69	194	65	130	129	227	32	76	183	65	194	119	140
70-79	97	33	65	65	114	16	16	114	13	117	59	71
≥80	33	10	21	22	37	6	5	38	8	35	16	27
p-value	0.04		0.02		0.52		0.03		0.41		0.06	
Gender												
Male	145	71	119	97	184	32	48	168	43	173	91	125
Female	179	37	97	119	194	22	49	167	43	173	103	113
p-value	0.03		0.01		0.07		0.02		0.04		0.05	
Marital status												
Married	205	76	140	141	249	32	60	221	56	225	121	160
Unmarried	119	32	76	75	129	22	37	114	30	121	73	78
p-value	0.02		0.04		0.06		0.05		0.03		0.07	
Education												
No formal education	76	32	43	65	86	22	16	92	13	95	38	70
Primary	119	43	81	81	140	22	35	127	30	132	67	95
Secondary	86	22	65	43	97	11	30	78	26	82	61	47
Higher secondary+	43	11	27	27	55	0	16	38	17	37	28	26
p-value	0.01		0.02		0.04		0.06		0.05		0.03	
Income												
<10,000	194	76	119	151	233	37	49	221	39	231	108	162
10,000-30,000	97	11	76	32	103	5	35	73	30	78	69	39
>30,000	33	21	21	33	42	12	13	41	17	37	17	37
p-value	0.04		0.03		0.02		0.01		0.02		0.04	
Chronic conditions												
0	86	22	65	43	86	22	0	108	0	108	43	65
1	119	43	81	81	140	22	30	132	26	136	67	95
≥2	119	43	70	92	152	10	67	95	60	102	84	78
p-value	0.06		0.05		0.03		0.02		0.01		0.06	
Self-efficacy												
Low	108	54	65	97	130	32	22	140	17	145	54	108
Medium	151	43	108	86	173	21	49	145	43	151	91	103
High	65	11	43	33	75	0	26	50	26	50	49	27
p-value	0.04		0.03		0.01		0.05		0.07		0.02	
Health literacy												
Inadequate	130	64	87	107	162	32	27	167	22	172	76	118
Marginal	108	32	70	70	130	10	38	102	35	105	65	75
Adequate	86	12	59	39	86	12	32	66	29	69	53	45
p-value	0.03		0.02		0.05		0.01		0.06		0.04	

**Table 5:** Distribution of sociodemographic characteristics and health-seeking behaviors (n = 432) Frequencies, percentages, and p-values are shown for relationships between variables and health-seeking behavior. p < 0.05 indicates a significant relationship.

Variable	Preventive screening		Doctor visits		ED visits		Hospitalization		Telehealth use		Traditional medicine	
	Yes	No	Yes	No	Yes	No	Yes	No	Yes	No	Yes	No
Age												
60-69	119	140	205	54	59	200	49	210	97	162	86	173
70-79	54	76	92	38	33	97	27	103	49	81	54	76
≥80	21	22	27	16	16	27	11	32	16	27	11	32
p-value	0.04		0.03		0.06		0.05		0.02		0.07	
Gender												
Male	86	130	157	59	60	156	49	167	76	140	81	135
Female	108	108	167	49	48	168	38	178	86	130	70	146
p-value	0.03		0.02		0.04		0.06		0.01		0.05	
Marital status												
Married	130	151	211	70	70	211	54	227	108	173	97	184
Unmarried	64	87	113	38	38	113	33	118	54	97	54	97
p-value	0.02		0.04		0.03		0.07		0.06		0.04	
Education												
No formal education	38	70	76	32	33	75	27	81	33	75	43	65
Primary	65	97	119	43	43	119	38	124	54	108	59	103
Secondary	54	54	86	22	22	86	16	92	43	65	38	70
Higher secondary+	37	17	43	11	10	44	6	48	32	22	11	43
p-value	0.01		0.06		0.05		0.04		0.03		0.02	
Income												
<10,000	108	162	194	76	76	194	65	205	86	184	108	162
10,000-30,000	65	43	86	22	22	86	16	92	54	54	32	76
>30,000	21	33	43	11	10	44	6	48	22	32	11	43
p-value	0.07		0.05		0.04		0.03		0.02		0.01	
Chronic conditions												
0	49	59	81	27	16	92	11	97	32	76	27	81
1	65	97	119	43	38	124	27	135	59	103	54	108
≥2	81	81	124	38	54	108	49	113	70	92	70	92
p-value	0.05		0.04		0.03		0.02		0.01		0.06	
Self-efficacy												
Low	54	108	108	54	38	124	27	135	43	119	54	108
Medium	86	108	146	48	43	151	38	156	65	129	65	129
High	54	22	70	6	27	49	22	54	54	22	32	44
p-value	<0.001		<0.001		0.001		<0.001		<0.001		0.001	
Health literacy												
Inadequate	59	135	130	64	54	140	38	156	59	135	70	124
Marginal	70	70	124	16	32	108	30	110	54	86	49	91
Adequate	65	33	70	28	22	76	19	79	49	49	32	66
p-value	<0.001		<0.001		0.005		0.001		<0.001		0.001	

Table 6 presents the results from multivariate logistic regression models examining factors associated with health behaviors. The key findings are as follows: Compared with males, females had 1.6 times greater odds of seeking health (AOR 1.6, 95% CI 1.1-2.5; p = 0.02) and 1.8 times greater odds of self-care (AOR 1.8, 95% CI 1.2-2.8; p = 0.006). Those with >5 years of education had 2.1 times greater odds of seeking health (AOR 2.1, 95% CI 1.1-4.2; p = 0.03) and 2.5 times greater odds of self-care (AOR 2.5, 95% CI 1.2-5.2; p = 0.01) than those with no education. High self-efficacy and adequate health literacy also positively predicted health behaviors after adjusting for confounders.

**Table 6:** Multivariate analysis of factors associated with health-seeking behaviors and self-care practices p < 0.05 indicates statistical significance.

Variable	Health-seeking behaviors		Self-care practices	
	AOR (95% CI)	p-value	AOR (95% CI)	p-value
Age (years)				
60-69	Ref	-	Ref	-
70-79	0.8 (0.5-1.2)	0.3	0.7 (0.4-1.1)	0.09
≥80	1.5 (0.8-2.7)	0.2	0.6 (0.3-1.2)	0.15
Female gender	1.6 (1.1-2.5)	0.02	1.8 (1.2-2.8)	0.006
Married	1.3 (0.8-2.1)	0.3	1.5 (0.9-2.4)	0.09
Education (years)				
0	Ref	-	Ref	-
1-5	1.3 (0.8-2.1)	0.3	1.6 (1.0-2.6)	0.04
>5	2.1 (1.1-4.2)	0.03	2.5 (1.2-5.2)	0.01
Income >Rs. 10,000	1.5 (1.0-2.3)	0.04	1.8 (1.2-2.8)	0.006
≥2 comorbidities	1.8 (1.2-2.8)	0.006	1.6 (1.1-2.4)	0.02
High self-efficacy	2.5 (1.5-4.2)	0.001	2.1 (1.3-3.5)	0.003
Adequate health literacy	1.8 (1.2-2.7)	0.004	2.0 (1.3-3.0)	0.001

Table 7 presents qualitative themes, subthemes, and quotations describing barriers such as limited healthcare access and a lack of preventive orientation, as well as facilitators such as social support and self-efficacy that impact health behaviors among older adults. The quotes provide insights into their lived experiences and perspectives in their own words.

**Table 7:** Qualitative themes on barriers and facilitators to self-care practices and health-seeking behaviors among older adults

Theme	Subtheme	Participant’s phrase
Limited healthcare access	Financial constraints	“I do not have money to see the doctor regularly.”
	Transportation barriers	“The hospital is very far and difficult to reach.”
	Long waits, rushed consultations	“Doctors do not have time to explain properly.”
	Ageism	“Younger patients get priority in hospitals.”
Preventive orientation	Lack of screening awareness	“I have never had cancer screening done before.”
	Focus on treatment	“I only see the doctor when I'm sick.”
	Reliance on home remedies	“I take traditional herbs instead of seeing a doctor.”
Social support	Family assistance	“My son takes me for medical appointments.”
	Spousal motivation	“My wife reminds me to take medicine daily.”
	Friends’ advice	“We all look out for each other’s health in our group.”
Self-efficacy	Information processing	“Healthcare instructions are difficult to understand.”
	Adherence motivation	“I lack the motivation to follow self-care advice.”
	Technique competency	“I cannot monitor my blood sugar properly.”

## Discussion

This study revealed that the majority of older slum residents had inadequate health literacy (45%) and self-efficacy (37.5%), resulting in significant gaps in health knowledge and self-care practices. Although three-fourths of the participants had a healthy diet and medication adherence, only 20% monitored their feet daily for diabetes complications. Only 45% of patients underwent recommended preventive cancer screening, and 75% of patients had at least two doctor visits a year, which seems insufficient given the high burden of chronic illness (75% with ≥1 condition).

The significant positive associations found between education, income, self-efficacy, and engagement in self-care align with prior evidence [20]. The poorer self-management seen among adults aged ≥80 years compared to 60- to 69-year-olds is consistent with age-related motivational decline, especially among vulnerable oldest-old slum dwellers focused on daily survival [21]. Greater functional impairment and entrenched dependence on family support likely erode self-agency within this subgroup.

Having ≥2 chronic conditions promoted foot care and glucose monitoring, likely due to greater perceived self-management needs in Asian cultures [22]. The persisting popularity of traditional medicine despite polypharmacy risks and outdated cultural health beliefs indicates deeply embedded attitudinal barriers to modern healthcare usage, meriting further study [23]. Low geriatric social security coverage [24] explains how financial constraints hamper preventive care orientation. In contrast to total self-reliance, family assistance improves diet and medication compliance. Lower self-efficacy among widowed older adults predicts self-neglect risks [25], necessitating targeted interventions promoting independence.

Overall, a suboptimal prevention orientation and secondary care overreliance rather than prudent self-care are problematic given inaccessible, unaffordable specialist services and hospital-related centrism in Indian healthcare [26]. Marginalized groups, particularly older women with mobility limitations and without family support networks, are especially vulnerable to net safety benefits in urban areas, unlike native villages [26]. Limited healthcare access due to affordability barriers, difficulties accessing clinics, long wait times, and perceived ageism emerged as key constraints on self-care and health-seeking behaviors among older adults. The lack of preventive orientation was evident in low awareness of preventive screenings, a greater focus on curative treatment, and a preference for home remedies over physician consultation. However, social support from family members, spouses, and friends facilitated adherence to self-care practices and medical consultations. Barriers such as difficulty understanding health instructions and a lack of motivation reduce self-efficacy for optimal self-care. These multilevel barriers need to be addressed through community-based educational interventions and capacity building to enable self-care agency among this marginalized group. Several previous studies that support these findings are as follows: A study conducted in Ghana in 2021 explored the perceived health and social care needs of older adults in two slums. The study showed that limited access to health and social care services in slums negatively impacts the quality of life of older adults. The study also revealed that social support from family members and friends facilitated adherence to self-care practices and medical consultations [27]. Another study conducted in 2022 explored the views of health professionals on the access to and use of health and social care services by slum-dwelling older adults. The study revealed that financial barriers, queues to access care services, attitudes of health professionals, long distances to health facilities, health illiteracy, and the unavailability of formal social care services were some of the factors that influenced access to and use of health and social care services by older slum dwellers [28]. A systematic review and qualitative meta-synthesis conducted in 2022 identified barriers to and facilitators of type 2 diabetes management among slum dwellers. This review revealed that individual, health system, and contextual factors influence type 2 diabetes management among slum dwellers. The review recommended that policymakers use the findings to reduce barriers and augment facilitators of type 2 diabetes management among slum dwellers [29]. A study conducted in 2023 explored barriers to healthcare utilization among patients with type 2 diabetes living in slums from the providers' perspective. The study revealed that financial barriers, a lack of trust in health care providers, and poor infrastructure were among the barriers to health care utilization among patients with type 2 diabetes living in slums. The study recommended that the healthcare system modify payment methods, improve patient-provider relationships, and increase the number of providers to improve healthcare utilization among slum dwellers [30].

Limitations

This study has certain limitations that should be considered when interpreting the results. First, the cross-sectional design provides evidence of associations but cannot confer causality regarding predictors of self-care and health-seeking behaviors. Second, the use of self-reported measures for key outcomes such as medication adherence may overestimate actual practices due to social desirability biases. The self-selected community-dwelling sample may indicate relatively better health than homebound older adults. Convenience sampling within selected slum clusters limits generalizability to the broader slum-dwelling population. The qualitative sample size of only 30 participants hindered saturation across diverse perspectives. The design effect was not considered in the study. The study measures lacked extensive reliability testing and multidimensional scales assessing self-care aspects such as problem-solving abilities. Residual confounding factors cannot be eliminated in multivariate analysis.

Recommendations

This study underscores key modifiable targets for improving self-care capacity and healthcare utilization among marginalized older adults through community-based programs. Longitudinal interventional studies implementing and evaluating health literacy and self-efficacy enhancement strategies such as peer counseling and m-health decision aids are essential next steps. Mixed methods implementation research can help refine grassroots eldercare models, addressing context-specific sociocultural barriers across urban slums. Robust measures, sampling techniques, and adequately powered qualitative samples increase the quality of the evidence. Cost-effectiveness analyses would assist advocacy efforts regarding social protection policies and geriatric-friendly service delivery models tailored for resource-limited settings with growing aging populations. Partnerships involving slum communities, health systems, local governments, and researchers can sustainably strengthen preventive behaviors, self-care skills, and healthcare access to promote active aging.

## Conclusions

This study contributes to the limited evidence base on promoting self-care and facilitating healthcare access among older adults in resource-poor urban settings with growing elderly populations. A multipronged strategy addressing individual, family, community, and health system barriers is essential to bridge gaps in preventive behaviors and self-management for this vulnerable yet often overlooked group. Supportive policies, elderly-friendly infrastructure, and healthcare capacity building to meet geriatric needs can potentially enhance self-care. Grassroots community health workers can improve health literacy and self-efficacy among marginalized slum elders through home visits, suitable advice, and resource access assistance. Gender-sensitive interventions prioritizing at-risk older women are urgently warranted to promote active aging.
